# Prodromal characteristics of dementia with Lewy bodies: baseline results of the MEMENTO memory clinics nationwide cohort

**DOI:** 10.1186/s13195-022-01037-0

**Published:** 2022-07-19

**Authors:** Frederic Blanc, Vincent Bouteloup, Claire Paquet, Marie Chupin, Florence Pasquier, Audrey Gabelle, Mathieu Ceccaldi, Paulo Loureiro de Sousa, Pierre Krolak-Salmon, Renaud David, Clara Fischer, Jean-François Dartigues, David Wallon, Olivier Moreaud, Mathilde Sauvée, Catherine Belin, Sandrine Harston, Anne Botzung, Timothée Albasser, Catherine Demuynck, Izzie Namer, Marie-Odile Habert, Stéphane Kremer, Olivier Bousiges, Marc Verny, Candice Muller, Nathalie Philippi, Geneviève Chene, Benjamin Cretin, Jean-François Mangin, Carole Dufouil

**Affiliations:** 1 CM2R (Memory Resource and Research Centre), Day Hospital, Geriatrics Department, University Hospital of Strasbourg, Strasbourg, France; 2 CNRS, ICube Laboratory, UMR 7357 and FMTS (Fédération de Médecine Translationnelle de Strasbourg), Team IMIS, University of Strasbourg, Strasbourg, France; 3 CHU de Bordeaux, Pôle de santé publique, Bordeaux, France; 4 Centre INSERM U1219, Institut de Santé Publique, d’Epidémiologie et de Développement (ISPED), Bordeaux School of Public Health, Université de Bordeaux, Bordeaux, France; 5 CM2R of Paris Nord, AP-HP, Groupe Hospitalier Saint-Louis Lariboisière Fernand Widal, Paris, France; 6 CATI Multicenter Neuroimaging Platform, Saclay, France; 7 INSERM U1171 and CM2R of Lille, CHRU de Lille, Hôpital Roger Salengro, University of Lille, Lille, France; 8 CM2R of Montpellier, CHU de Montpellier, Hôpital Gui de Chauliac, Montpellier, France; 9 CM2R of Marseille, CHU de Marseille, Hôpital La Timone, Marseille, France; 10 CM2R of Lyon, Hospices Civils de Lyon, Hôpital des Charpennes, Lyon, France; 11 CM2R of Nice, CHU de Nice, Institut Claude Pompidou, EA 7276 CoBTeK “Cognition Behaviour Technology”, Nice, France; 12 CM2R of Bordeaux, CHU de Bordeaux, Hôpital Pellegrin, Bordeaux, France; 13 CM2R of Rouen, Neurology Department, Rouen University Hospital, Rouen, France; 14 CM2R of Grenoble, CHU de Grenoble Alpes, Hôpital de la Tronche, Grenoble, France; 15 Memory Clinic, Hôpital Avicenne, AP-HP, Hôpitaux Universitaires, Paris-Seine-Saint-Denis, Bobigny, France; 16 CM2R of Bordeaux, CHU de Bordeaux, Hôpital Xavier Arnozan, Bordeaux, France; 17 CM2R Île-de-France Sud and Geriatrics Centre, Hôpital Pitié-Salpêtrière, AP-HP, Paris, France; 18 Université Pierre et Marie Curie et DHU FAST, UMR 8256 (CNRS), Paris, France; 19 NeuroSpin, I2BM, Commissariat à l’Énergie Atomique, Université Paris-Saclay, Saclay, France

**Keywords:** Dementia with Lewy bodies, Prodromal, Mild cognitive impairment, Subjective cognitive impairment, Lewy body disease, Mild neurocognitive impairment

## Abstract

**Background:**

Isolated subjective cognitive impairment (SCI) and mild cognitive impairment (MCI) are the prodromal phases of dementia with Lewy bodies (DLB). MEMENTO is a nationwide study of patients with SCI and MCI with clinic, neuropsychology, biology, and brain imaging data. We aimed to compare SCI and MCI patients with symptoms of prodromal DLB to others in this study at baseline.

**Methods:**

Participants of the French MEMENTO cohort study were recruited for either SCI or MCI. Among them, 892 were included in the Lewy sub-study, designed to search specifically for symptoms of DLB. Probable prodromal DLB diagnosis (pro-DLB group) was done using a two-criteria cutoff score among the four core clinical features of DLB. This Pro-DLB group was compared to two other groups at baseline: one without any core symptoms (NS group) and the one with one core symptom (1S group). A comprehensive cognitive battery, questionnaires on behavior, neurovegetative and neurosensory symptoms, brain 3D volumetric MRI, CSF, FDG PET, and amyloid PET were done.

**Results:**

The pro-DLB group comprised 148 patients (16.6%). This group showed more multidomain (59.8%) MCI with slower processing speed and a higher proportion of patients with depression, anxiety, apathy, constipation, rhinorrhea, sicca syndrome, and photophobia, compared to the NS group. The pro-DLB group had isolated lower P-Tau in the CSF (not significant after adjustments for confounders) and on brain MRI widening of sulci including fronto-insular, occipital, and olfactory sulci (FDR corrected), when compared to the NS group. Evolution to dementia was not different between the three groups over a median follow-up of 2.6 years.

**Conclusions:**

Patients with symptoms of prodromal DLB are cognitively slower, with more behavioral disorders, autonomic symptoms, and photophobia. The occipital, fronto-insular, and olfactory bulb involvement on brain MRI was consistent with symptoms and known neuropathology. The next step will be to study the clinical, biological, and imaging evolution of these patients.

**Trial registration:**

Clinicaltrials.gov, NCT01926249

## Introduction

The description of the prodromal phase of dementia with Lewy bodies (pro-DLB) is just emerging, in contrast to the description of the prodromal phase of Alzheimer’s disease [1]. There is nevertheless a consensus on the key symptoms of pro-DLB, combining rapid eye movement (REM) sleep behavior disorders (RBD), cognitive and alertness fluctuations, hallucinations, and parkinsonism [1]. The presence of 2 of the 4 key symptoms with mild cognitive impairment (MCI) indicates probable pro-DLB [1]. Beyond the key symptoms of the disease, other symptoms have been described as prodromal, such as autonomic dysfunction symptoms, including constipation or erectile dysfunction [2, 3], or behavioral symptoms such as depression [4, 5]. Delirium could also occur in the prodromal phase of DLB [6]. Prospective cohorts are needed in the context of pro-DLB to better determine the different characteristics of this disease [5, 7, 8].

Previous studies have described the cognitive pattern of pro-DLB. The MCI pattern was amnestic multidomain in 33 to 50% of patients, non-amnestic multidomain in 24 to 39%, and non-amnestic unidomain in 27 to 49% [9–11]. Cognitive disorders in pro-DLB patients include a predominant impairment of speed processing (particularly with Trail Making Test-A), attention, executive functions, and neurovisual functions [9–11]. Memory impairment is less frequent than in prodromal Alzheimer’s disease for verbal memory, whereas for visual memory, pro-DLB patients are frequently affected [10]. Language is most often unaffected, except for moments of fluctuation. Few studies exist on cerebrospinal fluid (CSF) in pro-DLB: the analysis of Tau, P-Tau, and Abeta-42 is normal in most cases [5, 12]. Reduced insular cortical thinning [13] and a decrease in gray matter concentration [14] have been demonstrated in pro-DLB patients using image processing on brain MRI T1 sequences. Pro-DLB patients have antero-superior insula atrophy when compared to healthy controls [13] and more preserved hippocampi when compared to pro-AD patients [1]. FP-CIT dopaminergic SPECT is a recognized biomarker of DLB. However, in the prodromal phase, this biomarker is not sufficiently sensitive: the value of FP-CIT in distinguishing probable pro-DLB from pro-AD is 61% for sensitivity and 89% for specificity [15].

In a large prospective cohort of patients attending memory clinics presenting either subjective cognitive impairment (SCI) and MCI patients, we undertook an ancillary study aiming to detect symptoms of DLB in a longitudinal framework. The aim of the present study was to compare MCI and SCI patients with symptoms of prodromal DLB to patients without any key symptom of DLB and patients with only one key symptom at the baseline for different characteristics: clinical, neuropsychological, cerebrospinal fluid (CSF), brain MRI, 18-fluoro-desoxy-glucose (FDG) PET, and amyloid PET one.

## Methods

The MEMENTO cohort is a clinic-based study aimed to investigate the evolution of a large variety of cognitive symptoms and subjective complaints over time, without any specific a priori hypothesis regarding the relationship with incident dementia. It was set up as an initiative of the French Plan Alzheimer 2008–2012 [16]. Recruitment took place within the French national network of university-based memory clinics (Centre Mémoire de Ressources et de Recherche [CM2R]). Among the 26 CM2Rs of the MEMENTO cohort (totaling 2323 patients), 12 CM2Rs agreed to become investigating centers for the Lewy MEMENTO sub-study [16]. Between April 2011 and June 2014, 892 patients consented to participate in the ancillary Lewy MEMENTO cohort over at least 4 years, including one visit per year.

### Study recruitment

Eligible adult participants for the MEMENTO study, and therefore for the Lewy MEMENTO sub-study, had to undergo at baseline all clinical examinations, brain MRI, and blood sampling. All participants had to have visual and auditory acuity compatible with neuropsychological testing and be affiliated to a health insurance scheme. The participants were screened for either light to moderate MCI or SCI, and they were recruited consecutively. Light MCI was defined as (1) performing worse than one standard deviation from the mean (compared to age and education norms) in one or more cognitive domains (memory, language, praxis, attention, executive functions, speed processing, visual-spatial abilities), this deviation being identified for the first time through cognitive tests performed recently, i.e., less than 6 months before the screening phase, and (2) having a Clinical Dementia Rating scale score ≤ 0.5 and being non-demented. A participant was eligible for inclusion in the isolated SCI stratum if he or she had SCI (assessed through visual analog scales) without any objective cognitive deficit as defined above and was aged 60 years or older. The battery of neuropsychological tests and detailed aspects of the inclusion/exclusion criteria were described previously for the MEMENTO cohort [16].

### Standard protocol approvals, registrations, and patient consents

All participants provided written informed consent for the MEMENTO cohort and the Lewy MEMENTO sub-study, and the protocol was approved by the ethics committee (Comité de Protection des Personnes Sud-Ouest et Outre Mer III). The protocol is registered in ClinicalTrials.gov (Identifier: NCT01926249, https://clinicaltrials.gov/ct2/show/NCT01926249).

### Study examinations

The baseline data collected at the memory clinic were described previously [16]. The Lewy MEMENTO sub-study included three additional sections added to the basic MEMENTO package. The first section focused on key symptoms of DLB: features of parkinsonism were evaluated using the Unified Parkinson’s Disease Rating Scale (UPDRS, part III), including akinesia, amimia, and rigidity (rated from 0 [no symptoms] to 4 [serious symptoms]) and falls. Fluctuations were assessed with the Mayo Clinic Fluctuations Scale [17] and the Newcastle-upon-Tyne Clinician Assessment of Fluctuation scale (CAF) [18]. The Hallucinations Parkinson’s Disease-Associated Psychotic Symptoms Questionnaire was used to evaluate the presence of hallucinations [19]. RBD was evaluated using a questionnaire based on the article by Gjerstad et al. [20] simplified into two questions for the patient and the caregiver, one concerning movements during sleep and the other concerning vivid dreams and nightmares. The second section focused on autonomic disorders and sensorineural symptoms and included: 5 min lying and standing blood pressure at 1, 2, and 3 min with a heart rate measurement; questionnaire on dry eyes, mouth, and nose; rhinorrhea, lacrimation, and salivation; constipation; libido and erectile dysfunction; and photophobia. Each item was rated from “no symptom = 0” to “daily symptom = 2.” The third section focused on neuropsychological aspects with the following sub-tests using the *Visual Object and Space Perception Battery (VOSP) allowing* the evaluation of visuo-perceptual and visuo-spatial abilities*:* “incomplete letters,” “position discrimination,” and “number location.” The DSM IV MINI500 test was used for the diagnosis of major depression (score ≥ 3).

### Definition of the groups

The pro-DLB group included patients with SCI or MCI and with at least two of the core features of DLB (fluctuations, RBD, hallucinations, and parkinsonism) during the first visit. The one symptom group (1S) included patients with only one key DLB symptom and the no symptom group (NS) patients without any core features of DLB. Each symptom was considered to be present as follows: for RBD, if the score was 1/2 or 2/2; for hallucinations, if sensation of passage, sensation of presence, illusions, non-visual and visual hallucinations, and delusion were detected; for fluctuations, if a cutoff score of 2/4 or over on the Mayo Clinic Fluctuations Scale was recorded (caregiver or patients); for parkinsonism, if one criterion among akinesia, rigidity, amimia, or falls was present. This description of each of the criteria is very close to the definition of the 2020 McKeith criteria with some subtle differences [1]. First, all types of hallucinations have been taken into account here including minor hallucinations (sensation of passage and sensation of presence) and non-visual hallucinations as well as delusion. Second, the parkinsonian syndrome was expanded to include the presence of amimia as well as the presence of falls.

### Imaging, blood, and cerebrospinal fluid

Neuroimaging acquisitions (brain MRI, 18F-FDG PET, amyloid PET) were coordinated by the Center for Automated Treatment of Images (CATI), a platform dedicated to multicenter neuroimaging [21]. The MRI and PET protocols as well as standardization and quality control procedures were described previously [16]. In our sample, brain MRI was available for 98.0% of participants (88% on a 3.0 T MRI scan, 1.5 T otherwise). FDG PET, amyloid-PET, and lumbar puncture were optional and were performed in respectively 68.6%, 30.8%, and 19.5% of participants.

At baseline, study-specific blood sampling included serum (12 tubes of 0.25 ml), plasma ethylenediaminetetraacetic acid (EDTA; 8 tubes of 0.25 ml), total blood heparin (2 tubes of 1 ml), plasma heparin (4 tubes of 500 μg), blood EDTA without plasma (1 tube of 0.25 ml), blood heparin without plasma (1 tube of 3 ml), and Tempus (2 tubes of 3 ml). Cerebrospinal fluid (CSF) was collected in polypropylene tubes following standardized conditions and using an atraumatic needle. Each CSF sample was transferred to the CSF bank within 4 h after collection and was centrifuged at 1000 × *g* at 4 °C for 10 min. CSF samples were aliquoted in polypropylene tubes (16 tubes of 250 μl) and stored at − 80 °C. All CSF and blood samples were stored in a centralized biobank (Genomic Analysis Laboratory-Biological Resource Centre [LAG-CRB], Pasteur Institut Lille, BB-0033-00071). LAG-CRB extracted genomic DNA from peripheral blood samples using Gentra Puregene blood kits (QIAGEN, Hilden, Germany). Apolipoprotein E (APOE) genotypes were determined by KBiosciences (Hoddesdon, UK; www.kbioscience.co.uk), using their own system of fluorescence-based competitive allele-specific polymerase chain reaction. Two APOE single-nucleotide polymorphisms, rs429358 and rs7412, allowed the identification of the three major APOE alleles (ε2, ε3, and ε4).

Measurements of CSF amyloid-β 42 peptide (Aβ_42_), CSF Aβ_40_, total tau, and phosphorylated tau (p-tau181) levels were done using the standardized commercially available INNOTEST sandwich enzyme-linked immunosorbent assay (Fujirebio, Ghent, Belgium).

### Data and statistics

The data presented are those obtained at the first Lewy MEMENTO visit. The mild cognitive impairment was defined following Petersen’s criteria [22]. Based on the results of the neuropsychological battery, we used a 1.5 SD cutoff (compared to age and education norms) to define impaired cognitive domains. Individuals not MCI were considered in isolated subjective cognitive impairment; cognitive complaints were documented through visual analog scales.

As previously described [23], we focused on 4 items of the Instrumental Activities of Daily Living (IADL) questionnaire (ability to use the telephone, mode of transport, responsibility for his own medication, and ability to handle finances) as a proxy of dependence. Blood pressure was measured in three steps: after 5 min of rest in the supine position and after 1 min and 3 min in the standing position. Hypotension was defined as a decrease of at least 20 mmHg between the supine position and the standing position for systolic blood pressure and 10 mmHg for diastolic blood pressure. We defined pathological levels of CSF biomarkers as follows: Abeta42, lower than 750 pg/mL; P-Tau, higher than 60 pg/mL; total Tau, higher than 350 pg/mL; and Abeta42/40, lower than 0.065 [24]. Brain MRI biomarkers of interest were hippocampal volume (mean of both hemispheres) obtained using the SACHA software [25], brain parenchymal fraction (BPF) computed from SPM8 software, and total white matter hyperintensities (WMH) using the WHASA software [26]. The mean and regional cortical thicknesses by hemisphere were obtained using FreeSurfer 5.3. Cortical sulcus modifications were studied: the average distance between the two walls of the pial surface was computed. This distance provides an estimation of the local atrophy leading the fold to open up [27]. The mean and regional FDG-PET singular uptake value ratios (SUVRs) were normalized to the cerebellum [28]. The amyloid PET (florbetapir or flutemetamol) pipeline of analysis was described previously [29].

Data are presented with numbers and percentages for qualitative variables and with medians and first and third quartiles for qualitative variables. Comparisons across subgroups were done using Fisher’s chi-square tests or Kruskal-Wallis tests, as appropriate. We presented overall tests for homogeneity and then post hoc pairwise comparisons. For the latter, a Bonferroni correction for test multiplicity was applied to *P*-values. Thereafter, for readability reasons, we then rescaled *P*-values to keep an *α* = 0.05 threshold for statistical significance.

We tested whether biomarkers of interest could differ according to the DLB subgroup. We computed logistic regressions for binary outcomes (i.e., abnormal CSF markers, amyloid PET positivity) and linear regressions for quantitative ones (MRI measures, FDG-PET SUVRs). We ran 1 model per biomarker. Associations of DLB subgroups on biomarkers were adjusted for age, gender, education, and APOE. For brain MRI markers, additional adjustment covariates were the type of MRI (manufacturer and magnet size), and intracranial volume (except for BPF). These adjustment covariates were chosen a priori and no subsequent selection was done. The NS group was considered as the reference category (vs 1S or pro-DLB). An OR > 1 indicates a higher probability of having an abnormal biomarker in the group of interest vs the NS group, as well as a beta > 0 indicates a higher level of the quantitative biomarker. Comparisons were considered statistically significant for *P*-values below *α* = 0.05. For FDG PET and MRI, when analyses were done at a regional level (a ROI or a sulcus), we used the false discovery rate (FDR) method to control the type I error rate.

Finally, we analyzed the incidence of dementia for each subgroup. The time period started at the first Lewy MEMENTO visit and ended at the date of dementia, death, or end of follow-up, whichever occurred first. Incidence rates were computed by the number of dementia cases divided by the sum of person-years of follow-up (given in 100 person-years). Probabilities of being not demented over time were described by a Kaplan-Meier curve, and groups were compared through a log-rank test.

Analyses were performed using the SAS version 9.4 software (SAS Institute, Cary, NC).

## Results

Among the 892 patients, 148 (16.6%) were in the Pro-DLB group, 275 (30.8%) were in the 1S group, and 469 (52.6%) were without any DLB core features (NS group). There were no differences between the 3 groups in terms of age, sex, education, and APoE4 genotype. The proportion of SCI and different types of MCI was different between the pro-DLB group and the NS group (*P* = 0.0052): there was less SCI (19.7% versus 27.4%), and more MCI, particularly more multidomain MCI (59.8% versus 41.9%) in the pro-DLB group than in the NS group.

### Presence of key symptoms

The demographic characteristics and cognitive and behavioral findings for the three groups are provided in Table 1. MMSE was lower in the pro-DLB group than in the NS group (*P* = 0.015), but not in the 1S group (*P* = 0.138). IADL were lower in the pro-DLB group than in the NS (*P* = 0.0014) and 1S (*P* = 0.043) groups.

**Table 1:** Characteristics of the Lewy MEMENTO groups. Prodromal dementia with Lewy bodies group (pro-DLB), group with only one core symptom (1S group), and group without any core DLB symptoms (NS group)

	NS group (***N*** = 469)		1S group (***N*** = 275)		Pro-DLB group (***N*** = 148)		Comparisons (***P***-values)			
							Global	NS vs 1S groups*	NS vs pro-DLB groups*	1S vs pro-DLB groups*
**Age, years**^**a**^	71.4	(66.1; 77.2)	71.7	(66.0; 77.7)	71.1	(63.1; 80.5)	0.98	1.00	1.00	1.00
**Gender, F/M**	298/171		163/112		94/54		0.48	0.74	1.00	1.00
**Education**^**b**^	57.4%		58.2%		47.6%		0.079	1.00	0.117	0.115
**APOE-4, %**	27.6%		25.6%		30.9%		0.53	1.00	1.00	0.78
**CDR = 0.5, %**	48.6%		47.6%		60.4%		**0.037**	1.00	0.037	0.061
**MMSE**^**a**^	29	(27; 30)	29	(27; 30)	28	(27; 29)	**0.021**	1.00	0.015	0.138
**IADL (*N*= 8) restriction,*N*(%)**							**0.0031**	1.00	0.0014	0.043
**No**	390	(84.2)	221	(81.5)	101	(70.1)				
**One**	47	(10.2)	33	(12.2)	24	(16.7)				
**At least two**	26	(5.6)	17	(6.3)	19	(13.2)				
**RBD**	0%		56.7%		75.0%		**NA**			
**Movements during sleep**	0%		49.8%		56.2%		**NA**			
**Nightmares, restless nights**	0%		20.1%		45.2%		**NA**			
**Hallucinations**	0%		13.8%		63.5%		**NA**			
**Passage hallucination**	0%		3.6%		26.7%		**NA**			
**Presence hallucination**	0%		4.0%		24.3%		**NA**			
**Visual hallucinations**	0%		2.5%		17.6%		**NA**			
**Auditory hallucinations**	0%		2.5%		12.8%		**NA**			
**Olfactory/taste hallucinations**	0%		3.6%		12.8%		**NA**			
**Visual illusion**	0%		2.5%		12.3%		**NA**			
**Delusion**	0%		1.1%		12.8%		**NA**			
**Parkinsonism**^**c**^	0%		24.4%		55.4%		**NA**			
**Facial expression (0/1/2/3/4)**	100/0/0/0/0		88.8/10.9/0.4/0/0		62.8/31.7/3.4/2.1		**NA**			
**Rigidity**	100/0/0/0/0		91.7/7.1/1.1/0/0		77.9/17.2/4.1/0.7/0		**NA**			
**Akinesia**	100/0/0/0/0		87.2/12.8/0/0/0		71.0/23.4/4.1/0.7/0.7		**NA**			
**Falls**	1.5%		3.3%		9.6%		**NA**			
**Fluctuations**	0%		5.1%		42.6%		**NA**			
**Drowsiness/lethargy**	10%		22.9%		53.4%		**NA**			
**Sleep > 2 h**	0.6%		3.6%		20.3%		**NA**			
**Staring into space**	0.4%		4.7%		18.4%		**NA**			
**Disorganized speech**	2.1%		5.8%		32.0%		**NA**			
**Memory complaint**^**a**^	5	(3.0; 6.0)	5	(3.0; 7.0)	6	(4.0; 7.0)	**0.0021**	1.00	0.0011	0.017
**Attentional complaint**^**a**^	4	(3.0; 6.0)	5	(3.0; 6.0)	6	(4.0; 7.0)	**0.0001**	0.62	< 0.0001	0.0003
**FCSRT sum of 3 free recall**^**a**^	30	(24.0; 35.0)	29	(24.0; 34.0)	28	(23.0; 34.0)	0.25	1.00	0.32	0.48
**Fluency letter P**^**a**^	21	(16.0; 26.0)	21	(17.0; 26.0)	20	(14.0; 25.0)	0.15	1.00	0.23	0.22
**Fluency animals**^**a**^	29	(23.0; 35.0)	28	(22.0; 34.0)	27	(20.0; 32.0)	**0.0065**	0.17	0.0093	0.49
**Rey figure****3****min recall**^**a**^	18	(11.0; 23.0)	16.5	(10.0; 21.0)	16	(10.3; 22.0)	0.128	0.21	0.49	1.00
**TMT A s/good move**^**a**^	1.7	(1.4; 2.3)	1.8	(1.3; 2.3)	1.9	(1.5; 2.5)	**0.030**	1.00	0.040	0.054
**TMT B s/ good move**^**a**^	3.6	(2.6; 5.0)	3.6	(2.7; 5.1)	4.2	(2.8; 5.6)	0.074	1.00	0.074	0.20
**VOSP position discrimination**^**a**^	20	(19.0; 20.0)	20	(19.0; 20.0)	20	(19.0; 20.0)	**0.0022**	1.00	0.0015	0.045
**VOSP number location**^**a**^	9	(8.0; 10.0)	10	(9.0; 10.0)	9	(9.0; 10.0)	0.68	1.00	1.00	1.00
**VOSP fragmented letters**^**a**^	20	(19.0; 20.0)	20	(19.0; 20.0)	20	(19.0; 20.0)	**0.041**	1.00	0.041	0.129
**Cognitive profile,*N*(%)**	.		.		.		**0.0003**	0.92	0.0052	0.092
**SCI**	125	(27.4)	69	(25.6)	28	(19.7)				
**Pure a-MCI**	26	(5.7)	30	(11.1)	12	(8.5)				
**Multidomain a-MCI**	135	(29.6)	83	(30.7)	55	(38.7)				
**Pure na-MCI**	114	(25.0)	60	(22.2)	17	(12.0)				
**Multidomain na-MCI**	56	(12.3)	28	(10.4)	30	(21.1)				
**NPI-C ≥ 1 depression,*N*(%)**	32.5%		36.9%		49.2%		**0.0032**	0.71	0.0024	0.075
**MINI 500 depression,*N*(%)**	6.7%		11.1%		26.2%		**< 0.0001**	0.12	< 0.0001	< 0.0001
**NPI-C ≥ 1 anxiety,*N*(%)**	43.9%		45.8%		61.0%		**0.0033**	1.00	0.0029	0.018
**NPI-C ≥ 1 apathy,*N*(%)**	17.8%		25.1%		36.1%		**< 0.0001**	0.071	< 0.0001	0.086

*Abbreviations*: *a-MCI* amnestic mild cognitive impairment, *ATD* antidepressant, *ChEI* cholinesterase inhibitor, *CSF* cerebrospinal fluid, *Dopa=* L-Dopa, *FAB* Frontal Assessment Battery, *MMSE* Mini-Mental Status Examination, *na-MCI* non-amnestic MCI, *NL* neuroleptics, *N* number, *RBD* rapid eye movement sleep behavior disorder, *SCI* subjective cognitive impairment, *TMTA* Trail Making Test A *TMTB*, Trail Making Test B, *UPDRS* Unified Parkinson’s Disease Rating Scale

^a^Median (Q1 = first quartile − Q3 = third quartile)

^b^School leaving certificate; percentage

^c^As rated on UPDRS, out of 4

*P-values for post hoc pairwise comparisons were adjusted with Bonferoni correction and then rescaled to keep an *α* = 0.05 statistically significant threshold for readability reason

Within the pro-DLB group, 103 (69.6%) patients had two core features of DLB, 36 (24.3%) had three core features, and 9 (6.1%) had four core features. The frequency of core symptoms in the pro-DLB group was as follows: 42.6% fluctuations, 75% RBD, 63.5% hallucinations, and 55.4% parkinsonism. The most frequent symptoms of hallucinations in the Pro-DLB group were passage sensation (26.7% of patients), presence sensation (24.3%), and well-formed visual hallucinations (17.6%). Other psychotic symptoms were distributed as follows: auditory hallucinations in 12.8%, olfactory or taste hallucinations in 12.8%, delusion in 12.8%, and visual illusions in 12.3%. For parkinsonism, the most frequent symptom in this group was amimia, found in 37.2% of patients. On the Mayo Clinic Fluctuations Scale, the two items most frequently present in the pro-DLB group were drowsiness (53.4% of patients) and cognitive fluctuations expressed through changes in thought or language (32.0%). The CAF score was abnormal in 21.7% of patients in the pro-DLB group, 7.6% of patients in the 1S group, and 2% in the NS group.

### Autonomic and sensorineural symptoms

Autonomic symptoms were more frequent in the pro-DLB group than in the other two groups (Table 2). The most frequent in the pro-DLB group were sicca syndrome (especially dry mouth, 43.8%), constipation (34.7%), sexual dysfunction (including decreased libido or erectile dysfunction, 32.8%), and rhinorrhea (27.9%). Photophobia was quite frequent in this group (36.3%). Dry mouth was more frequent in the pro-DLB group than in the 1S (*P* = 0.009) and NS (*P* < 0.0001) groups, and more frequent in the 1S group than in the NS group (*P* = 0.0024). Eye dryness was more frequent in the pro-DLB group than in the NS group (*P* = 0.0014), and more frequent in the 1S group than in the NS group (*P* = 0.0005). Hypersalivation was more frequent in the pro-DLB group than in the NS (*P* < 0.0001) and 1S (*P* = 0.0049) groups. Lacrimation was more frequent in the pro-DLB (*P* < 0.0001) and 1S (*P* = 0.0032) groups than in the NS group. Rhinorrhea was more frequent in the pro-DLB group than in the NS (*P* < 0.0001) and 1S (*P* = 0.0008) groups. Constipation was more frequent in the pro-DLB group than in the NS (*P* < 0.0001) and 1S (*P* = 0.0012) groups. Sexual dysfunction frequency was globally different between the groups, but there was no significant difference when we did a group-to-group analysis. There was no difference in the cardiovascular metrics between the pro-DLB, 1S, and NS groups.

**Table 2:** Autonomic and sensorineural symptoms in the prodromal dementia with Lewy bodies patients’ group (pro-DLB group), compared to the group with only one core feature (1S) and the group without any DLB core feature (NS)

	NS group (***N*** = 469)		1S group (***N*** = 275)		Pro-DLB group (***N*** = 148)		Comparisons (***P***-values)			
							Global	NS vs 1S groups*	NS vs pro-DLB groups*	1S vs pro-DLB groups*
**Dry mouth,*N*(%)**	84	(18.5)	80	(29.3)	64	(43.8)	< 0.0001*	0.0024	< 0.0001	0.009
**Eye dryness,*N*(%)**	54	(11.9)	62	(22.7)	35	(24.0)	< 0.0001*	0.0005	0.0014	1
**Nasal dryness,*N*(%)**	26	(5.8)	31	(11.4)	27	(18.5)	< 0.0001*	0.023	< 0.0001	0.137
**Hypersalivation,*N*(%)**							< 0.0001*	0.16	< 0.0001	0.0049
Occasional hypersalivation	11	(2.4)	16	(6.0)	20	(13.8)				
Daily hypersalivation	4	(0.9)	1	(0.4)	4	(2.8)				
**Lacrimation,*N*(%)**							< 0.0001*	0.0032	< 0.0001	0.23
Occasional lacrimation	35	(7.6)	41	(15.4)	26	(18.2)				
Daily lacrimation	3	(0.7)	3	(1.1)	7	(4.9)	.			
**Rhinorrhea,*N*(%)**							< 0.0001*	0.62	< 0.0001	0.0007
Occasional rhinorrhea	38	(8.3)	28	(10.3)	29	(19.7)				
Daily rhinorrhea	9	(2.0)	8	(3.0)	12	(8.2)				
**Photophobia,*N*(%)**							< 0.0001*	0.015	< 0.0001	0.008
Occasional photophobia or with certain types of light	61	(13.2)	58	(21.3)	44	(30.1)				
Permanent photophobia or with all types of light	7	(1.5)	5	(1.8)	9	(6.2)				
**Constipation,*N*(%)**							< 0.0001*	0.19	< 0.0001	0.0012
Occasional constipation but not requiring treatment	47	(10.2)	46	(16.9)	26	(17.7)				
Frequent constipation requiring the use of laxatives	22	(4.8)	11	(4.0)	25	(17.0)				
**Sexual dysfunction,*N*(%)**							< 0.0001*	0.061	0.051	1
Slightly modified	52	(11.6)	27	(10.5)	20	(14.6)				
Severely modified	21	(4.7)	31	(12.1)	25	(18.2)				
**SBP mmHg (Q1–Q3)**^**a**^	133.5	(122.3; 147.0)	130.5	(118.7; 144.7)	135.3	(119.3; 149.3)	0.144	0.17	1	0.55
**DBP mmHg (Q1-Q3)**^**a**^	77.2	(69.3; 84.0)	76	(69.0; 84.0)	77.3	(69.7; 86.0)	0.50	1	1	0.73
**HR bpm (Q1––Q3)**^**a**^	70.7	(64.7; 79.3)	70.7	(64.3; 79.7)	72	(66.0; 80.0)	0.49	1	0.7	1
**Systolic hypotension,*N*(%)**	39	(9.4)	36	(14.5)	20	(16.0)	0.052	0.147	0.123	1
**Diastolic hypotension*N*(%)**	29	(7.0)	26	(10.4)	13	(10.4)	0.24	0.37	0.66	1
**Variation in HR at orthostatism in bpm (Q1–Q3)**^**a**^	7	(3.0; 12.0)	7	(4.0; 12.0)	8	(5.0; 12.0)	0.134	0.39	0.27	1

*Abbreviations*: *Bmp* beats per minute, *DBP* diastolic blood pressure (diastolic hypotension = if DBP decreased by more than 10 mmHg), *HR*, heart rate, *SBP* systolic blood pressure (systolic hypotension = if SBP decreased by more than 20 mmHg)

^a^Median (Q1 = first quartile − Q3 = third quartile)

*P-values for post hoc pairwise comparisons were adjusted with Bonferoni correction and then rescaled to keep an *α* = 0.05 statistically significant threshold for readability reason

### Cognitive, functional, and behavioral results

Overall, our results depict a more impaired cognitive, functional, and behavioral profile in the pro-DLB group than in the other two groups (Table 1). The median MMSE was slightly lower in the pro-DLB group compared to the NS group (*P* = 0.015). The cognitive complaint was more severe in the pro-DLB group, whether at the attention level (versus NS group, *P* < 0.0001; versus 1S group, *P* < 0.0003) or memory level (versus NS group, *P* = 0.0011; versus 1S group, *P* = 0.017). A SCI profile was observed in 19.7% of pro-DLB, 25.6% of 1S, and 27.4% of NS patients. On the other hand, the multidomain amnesic MCI was more frequent in the pro-DLB group (38.7%) than in the NS or 1S groups (29.6% and 30.7%, respectively), as well as multidomain non-amnestic MCI (21.1% in the pro-DLB group vs 12.3% for NS and 10.4 for 1S groups). The cognitive profile in the pro-DLB group was for most patients multidomain. Semantic fluency (*P* = 0.0093), Trail Making Test A (TMT-A, *P* = 0.040), fragmented letters (*P* = 0.041), and position discrimination (*P* = 0.0015) of the Visual Object and Space Perception (VOSP) battery were more deficient in the pro-DLB group than in the NS group, and VOSP position discrimination (*P* = 0.045) was lower in the pro-DLB group than in the 1S group (Table 1).

The scores on the functional scales were also worse in the pro-DLB group: the CDR 0.5 was more frequent (60.4%) than in the NS group (48.6%; *P* = 0.037) but not in the 1S group (47.6%; *P* = 0.061); a restriction of IADL was found in 29.1% of patients in the prodromal group, compared to 18.5% in the 1S group (*P* = 0.043), and 15.8% in the NS group (*P* = 0.0014).

On the NPI-C, a higher proportion of patients in the pro-DLB group presented symptoms of anxiety compared to the 1S (*P* = 0.018) and NS (*P* = 0.0029) groups, apathy compared to the NS group (*P* < 0.0001) but not the 1S group (*P* = 0.086), and depression compared to the NS group (*P* = 0.0024) but not the 1S group (*P* = 0.075). According to the MINI 500, 26.2% of the pro-DLB group could be considered to have major depression (*P* < 0.0001), significantly higher than in both the 1S and NS groups (*P*-values < 0.0001).

### Genetic, Alzheimer’s biomarkers, and FDG PET results

**Fig. 1:**
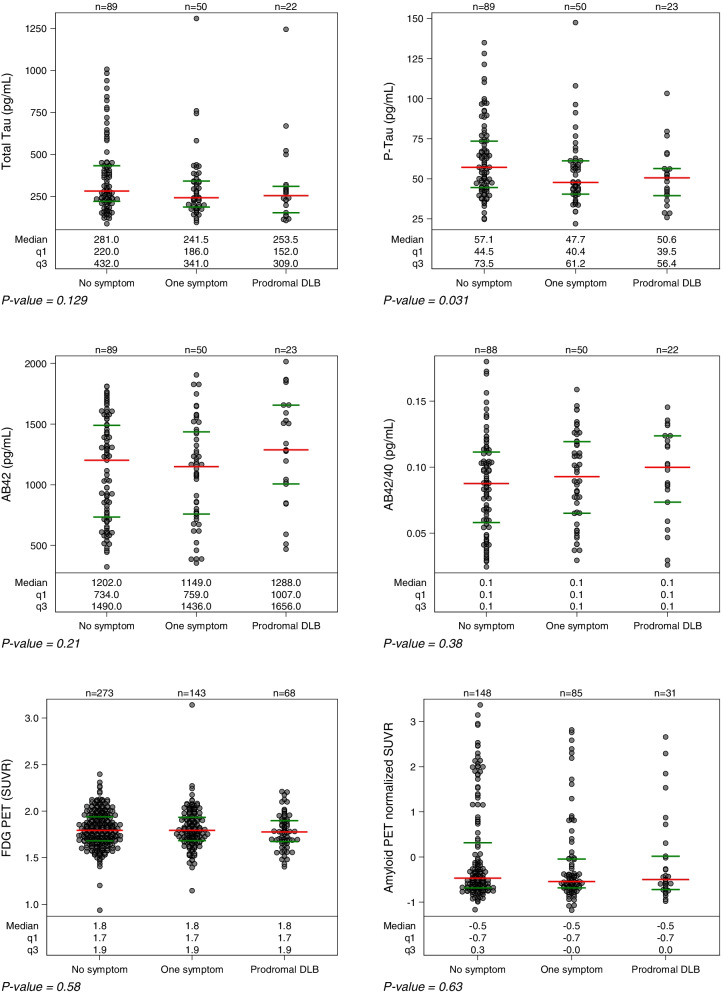
CSF, FDG PET, and amyloid PET analysis of the three groups: pro-DLB group (2 or more core symptoms of DLB), one symptom group (one core symptom), and no symptom group (no core symptom). For descriptive purposes, amyloid PET SUVRs were standardized for each radioligand

**Fig. 2:**
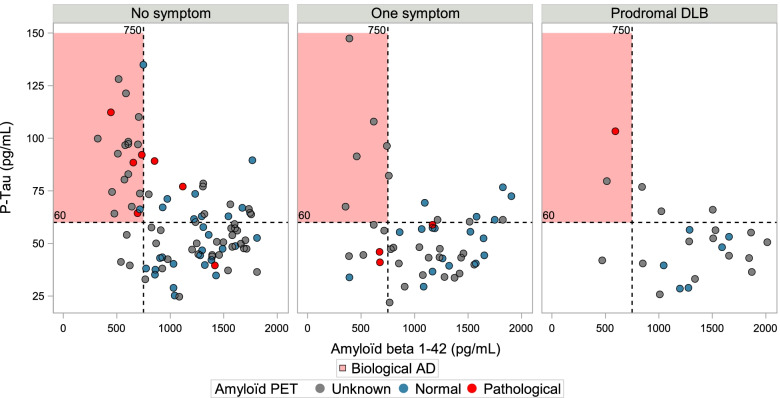
Results of the CSF and amyloid PET biomarkers for prodromal dementia with Lewy bodies group (pro-DLB), group with only one core symptom (1S), and group without any core DLB symptoms (NS)

The acceptance rates were comparable across the groups for all examinations but lower in the pro-DLB group for amyloid PET (21%, *P* = 0.04). In terms of genetic aspects, no difference was found for ApoE4 status (*P* = 0.53; Table 1). The CSF biomarker analysis showed that the pro-DLB group had lower P-Tau (50.6 versus 57.1 pg/ml for the NS group; *P* = 0.031) but this was not significant after adjustment for sex, age, education level, and ApoE (Fig. 1, Table 3, *P* = 0.15). The proportion of patients with abnormal CSF biomarkers was significantly higher for P-Tau and Tau in the NS group (Fig. 2). PET amyloid was positive in 36/149 (24%), 15/84 (17.9%), and 6/31 (19.4%) for the NS, 1S, and pro-DLB groups, respectively. The local and global analysis did not find any differences between the groups for FDG PET (*P* = 0.59) or amyloid PET (*P* = 0.51).

**Table 3:** Associations between the three groups of patients and biomarkers: prodromal dementia with Lewy bodies group (pro-DLB), group with one core symptom (1S group), group without any core DLB symptoms (NS group)

	1S (vs NS)		Pro-DLB (vs NS)		***P***-value
	OR	[IC95%]	OR	[IC95%]	
ABeta42 abnormal (vs normal)	0.99	[0.36; 2.71]	0.25	[0.04; 1.61]	0.33
T-Tau abnormal (vs normal)	0.36	[0.15; 0.91]	0.40	[0.10; 1.66]	0.067
P-Tau abnormal (vs normal)	0.48	[0.21; 1.08]	0.48	[0.14; 1.64]	0.15
ABeta42/40 abnormal (vs normal)	0.66	[0.25; 1.71]	0.86	[0.20; 3.66]	0.69
Amyloid PET + (vs −)	0.88	[0.40; 1.93]	1.13	[0.36; 3.58]	0.91
	**Beta**^a^	**[IC95%]**	**Beta**^a^	**[IC95%]**	***P*-value**
Intracranial volume, cm^3^	− 5.55	[− 22.4;11.28]	− 14.3	[− 35.6; 7.01]	0.41
BPF, %	− 1.11	[− 1.85; − 0.36]	− 0.42	[− 1.36; 0.53]	**0.015**
Hippocampal volume, mm^3^	0.02	[− 0.14; 0.17]	− 0.09	[− 0.29; 0.11]	0.61
Mean cortical thickness, mm	− 0.01	[− 0.02; 0.01]	− 0.01	[− 0.03; 0.01]	0.36
ROI cortical thickness ROI, mm	− 0.01	[− 0.03; 0.01]	− 0.02	[− 0.05; 0.01]	0.26
WMH volume, log (mm^3^)	0.15	[ 0.01; 0.29]	0.11	[− 0.07; 0.29]	0.094
SUVR PET-FDG	0.02	[− 0.02; 0.06]	− 0.02	[− 0.07; 0.03]	0.43
SUVR PET-FDG for disease specific ROI	− 0.00	[− 0.05; 0.04]	− 0.03	[− 0.09; 0.04]	0.68

The models in the table are adjusted for sex, age, education level, ApoE, type of MRI (if MRI measurement), and intracranial volume (if MRI and except for intracranial volume and BPF)

*Abbreviations*: *ADNI* Alzheimer’s Disease Neuroimaging Initiative, *BPF* brain parenchymal fraction, *FDG* fluorodeoxyglucose, *PET* positron emission tomography, *ROI* region of interest, *WMH* white matter hypersignals

^a^For a change in 1 unit of the biomarker

### MRI results

**Fig. 3:**
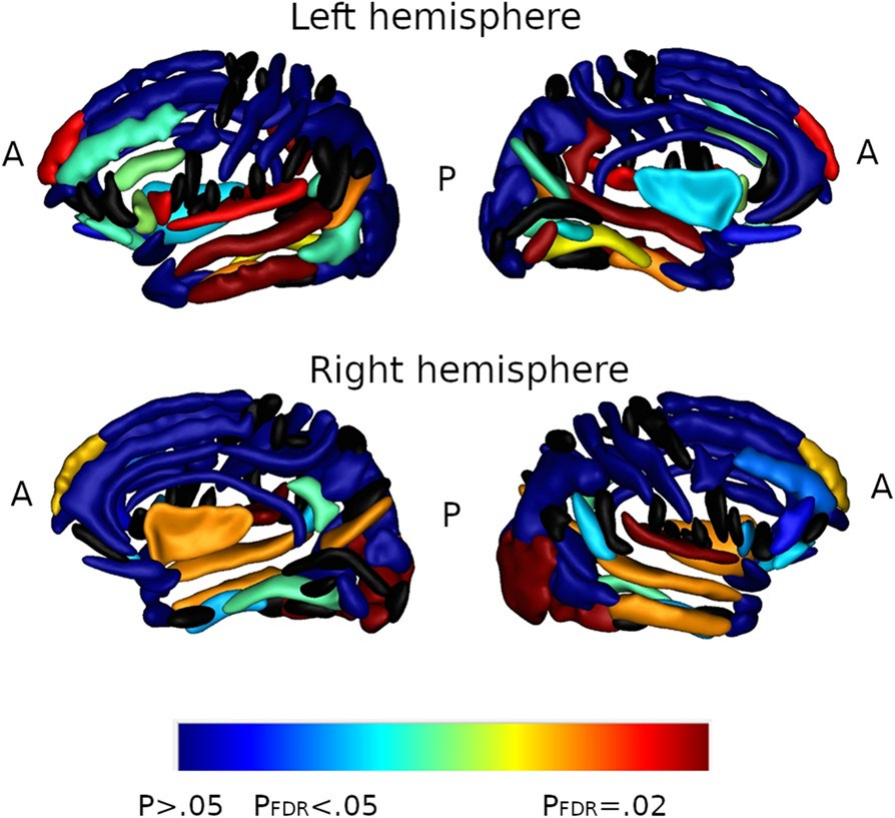
Enlargement of brain sulci in patients with one (1S group) or two (pro-DLB) core symptoms of prodromal dementia with Lewy bodies

Among 892 patients, 874 (98%, comparable across groups) patients had a brain MRI. No difference was found for hippocampal volume (*P* = 0.61), total intracranial volume (*P* = 0.41), mean cortical thickness (*P* = 0.36), ROI cortical thickness (*P* = 0.26), or white matter hypersignals volume (*P* = 0.094) (Table 3). The BPF was significantly different across groups (*P* = 0.015, Table 3), but the decrease was observed only in the 1S group (*P* = 0.004). Focal cortical thickness analysis found a lower cortical thickness in the left fusiform (*P* = 0.0088) and right superior temporal (*P* = 0.024) gyri in the pro-DLB group, but not if FDR-corrected. Sulcus-based brain MRI analysis, FDR-corrected, showed fold opening of different sulci combining the fronto-insular, occipital, temporal, and olfactory regions in the 1S and pro-DLB groups (Fig. 3).

### Progress towards dementia

During the follow-up after the first Lewy MEMENTO visit (median follow-up, 2.6 years; 1st quartile, 2.0 years; 3rd quartile, 3.1 years), the rate of progression per 100 patient-years was 1.7 [CI95%, 0.98–2.71] for the NS group (34 patients), 1.6 [CI95%, 0.71–2.97] for the 1S group (18 patients), and 1.7 [CI95%, 0.54–3.90] for the pro-DLB group (12 patients). We found no difference between the 3 groups regarding evolution to all-cause dementia (log-rank test *P* = 0.99).

## Discussion

We describe here a large nationwide study that specifically addressed the prodromal phase of dementia with Lewy bodies, at the baseline of the study. Clinically, patients in the pro-DLB group had 75% of RBD; 63.5% of hallucinations, particularly passage and presence hallucinations; and 55.4% of parkinsonism. Among fluctuations, drowsiness and cognitive fluctuations were frequent in the pro-DLB group. The most frequent symptom of parkinsonism in the pro-DLB group was amimia. Dry mouth, constipation, and rhinorrhea were the most frequent autonomic symptoms and photophobia was rather frequent, in the pro-DLB group when compared to the 1S and NS groups. Pro-DLB patients usually had a multidomain cognitive profile, with attentional and memory complaints. Concerning behavior, the Pro-DLB group had an over-representation of depression and anxiety. Cross-sectional brain imaging analysis showed a global decrease in brain volume (BPF) in the 1S group and a fold opening of occipital, olfactory, temporal, and fronto-insular regions in the 1S and pro-DLB groups. These results might suggest that a weak and localized atrophy process could be in progress.

### Clinical issues

The proportion of patients with probable pro-DLB in this study (16.6%) is consistent with the proportion of DLB in neuropathological studies, where it is between 4 and 24.7% of demented people [30]. Seventy-five percent of patients of the pro-DLB group in our study presented RBD, the same proportion as in autopsy-confirmed DLB demented patients [31]. Asking about hallucinations at an early stage makes it possible to have a description of them not by relatives but by the patients themselves. The most frequent symptoms were passage (26.7% of patients) and presence (24.3%) of hallucinations, which is consistent with a previous study on MCI patients [32]. Globally, 63.5% of the pro-DLB group had hallucinations and 31% had at least two types of hallucinations. Pro-DLB patients had a delusion in 12.8%. Delusion was previously described as a way of entering in the disease [33].

We found symptoms of anxiety and depression respectively in 61% and 49.2% and a diagnosis of major depression in 26.2%. In this connection, a Japanese study reported that 14% of people aged over 50 years hospitalized for depression had prodromal or demented DLB [4]. For fluctuations, more than half of the pro-DLB group reported drowsiness, before cognitive fluctuations. The scales for fluctuations detected fluctuations in the pro-DLB group in 21.7% of patients with CAF and 42.6% with the Mayo Clinic Fluctuations Scale. Using the latter scale, such fluctuations were previously reported in 76% of pro-DLB cases [10]. Parkinsonism was very discreet in our pro-DLB patients. A decrease in facial expression was the most common motor symptom in our study. This is consistent with a study of 26 prodromal DLB patients that found this to be an early symptom [8].

Autonomic dysfunction symptoms increased in parallel with DLB symptoms. Sicca syndrome was the autonomic dysfunction symptom most frequently described by patients, twice as many as in the NS group. To our best knowledge, this has never been described before in prodromal DLB or even in DLB. Sicca syndrome is a key symptom of primary Sjogren’s syndrome during which dementia could appear [34]. In contrast, facial secretion symptoms, such as rhinorrhea, lacrimation, and salivation, were also frequently found in our study. In prodromal DLB increased saliva was previously described [35, 36] unlike rhinorrhea and lacrimation. Among the increases in facial secretion, rhinorrhea was the most frequent in the pro-DLB group while being statistically different from the other two groups. The frequency of constipation is rather heterogeneous depending on the studies and countries: a study in the general population in France previously found a frequency of 22.4% [37]. In the pro-DLB group, we found a frequency of 34.7% against 15% in the NS group. This symptom has been described as occurring 9.3 years before the dementia phase of DLB [2]. Erectile dysfunction is an early symptom of DLB [8]. In our study, the frequency of sexual dysfunction (including erectile dysfunction but also a decrease or abolition of libido) in the pro-DLB group was 32.8%. In practice, autonomic disorders should therefore be systematically looked for in a context of mild cognitive disorder, particularly in the case of sicca syndrome, constipation, and rhinorrhea. The description of photophobia in Parkinson’s disease was recently done but not in DLB [38]. In our study, the higher the number of core symptoms of DLB present, the higher the frequency of photophobia, with a maximum of 36.3% in the pro-DLB group.

Globally, the 1S group could represent a possible pro-DLB group, and interestingly, the proportion of usual symptoms of DLB was intermediate between the NS group and the probable pro-DLB group: this was the case for autonomic, sensorineural, and behavioral symptoms. The 1S group therefore could correspond to an intermediate phase.

### Cognitive profile and outcome

The cognitive profile of the pro-DLB group was more multidomain, non-amnestic and amnestic, and less SCI than the NS and 1S groups. This is consistent with previous studies [9, 10, 39]. The main characteristics found in the pro-DLB group were low speed processing, low semantic fluency, and visuoperceptual and visuo-spatial impairment (Table 1). The evolution to dementia of patients during the very first years of follow-up was not different between the 3 groups, with few patients who had progressed to dementia.

### Biomarkers

For CSF, the proportion of patients with abnormal Alzheimer’s biomarkers was higher in the NS group than in the other two groups, but no difference was found for ABeta42 and ABeta42/40, and no difference was found for amyloid PET. These results are consistent with a previous study [12] and argue in favor of the absence of tangles in the pro-DLB group. However, the number of subjects tested was low, representing less than one-third of the cohort.

### Brain MRI and FDG PET

At the global and focal levels, there was no difference between the groups for FDG PET. On the contrary, brain MRI showed a smaller BPF in the 1S group. Moreover, the widening of sulci including fronto-insular sulci, occipital, temporo-occipital sulci, and olfactory sulci (FDR corrected) in the 1S and pro-DLB groups is of high interest. First, the modifications in the olfactory sulci are highly consistent with neuropathology, where patients even with incidental Lewy bodies had these lesions in the olfactory bulbs [40]. Second, the fronto-insular involvement in the prodromal phase of DLB has been demonstrated previously, particularly in the anterior part of the insula [13, 41]. Third, the temporo-occipital involvement is of interest regarding hallucinations since these regions are in the ventral stream of the visual pathway (the “what” pathway) devoted to visual gnosis.

### Limitations

Even if we used different biomarkers, including CSF, amyloid PET, FDG PET, and MRI, no specific biomarker for DLB, such as FP-CIT dopaminergic imaging, was used in our study [15]. The questionnaire used for RBD might be too sensitive; however, the high proportion of RBD in our pro-DLB cohort is quite consistent with previous studies [8, 31].

Another limitation is our sample might not be representative of the full pro-DLB, since we only have individuals attending memory clinics. Again, longitudinal data will partly address this question while it will allow exploring the different patterns of evolution in our sample from early to late DLB individuals.

Finally, we acknowledge the absence of selected comparison groups like healthy controls or Alzheimer’s disease individuals. Although this methodological approach could give interesting results, it is well known that it maximizes the differences across the subgroups and then could lead to non-reproducible results in a clinical setting. We assume that the MEMENTO cohort sample is more representative of the population attending memory clinics, and then our work gives more robust results.

## Conclusion

This study provides a description of a nationwide cohort of pro-DLB patients at the baseline of the study. We have reported new, frequently occurring symptoms in this group of patients: sicca syndrome, photophobia, lacrimation, and rhinorrhea. The cognitive profile, biomarkers, and PET results are consistent with the literature. Changes in the brain sulci, particularly olfactory and insula sulci, are also consistent with both neuropathological and other cohort data. We did not find any difference in terms of progression to dementia during the very first years of follow-up. The next step will be to investigate in detail the clinical, biological, and imaging evolution of these patients.

## Data Availability

The datasets used and analyzed during the current study are available from the corresponding author on a reasonable request.

## Abbreviations

APOE: Apolipoprotein E
CSF: Cerebrospinal fluid
BPF: Brain parenchymal fraction
DLB: Dementia with Lewy bodies
FDR: False discovery rate
FDG: 18-Fluoro-desoxy-glucose
MCI: Mild cognitive impairment
REM: Rapid eye movement
RBD: REM sleep behavior disorders
SCI: Subjective cognitive impairment
WMH: White matter hyperintensities

## References

[1] McKeith IG, Ferman TJ, Thomas AJ, Blanc F, Boeve BF, Fujishiro H, Kantarci K, Muscio C, O’Brien JT, Postuma RB (2020). Research criteria for the diagnosis of prodromal dementia with Lewy bodies. Neurology.

[2] Fujishiro H, Iseki E, Nakamura S, Kasanuki K, Chiba Y, Ota K, et al. Dementia with Lewy bodies: early diagnostic challenges. Psychogeriatrics. 2013;13(2):128-38. 10.1111/psyg.12005 23909972

[3] Postuma RB, Iranzo A, Hu M, Hogl B, Boeve BF, Manni R, Oertel WH, Arnulf I, Ferini-Strambi L, Puligheddu M (2019). Risk and predictors of dementia and parkinsonism in idiopathic REM sleep behaviour disorder: a multicentre study. Brain.

[4] Takahashi S, Mizukami K, Yasuno F, Asada T (2009). Depression associated with dementia with Lewy bodies (DLB) and the effect of somatotherapy. Psychogeriatrics.

[5] van de Beek M, van Steenoven I, van der Zande JJ, Barkhof F, Teunissen CE, van der Flier WM, Lemstra AW (2020). Prodromal dementia with Lewy bodies: clinical characterization and predictors of progression. Mov Disord.

[6] Sunwoo MK, Hong JY, Choi J, Park HJ, Kim SH, Lee PH (2013). α-Synuclein pathology is related to postoperative delirium in patients undergoing gastrectomy. Neurology.

[7] Bousiges O, Philippi N, Lavaux T, Perret-Liaudet A, Lachmann I, Schaeffer-Agalede C, Anthony P, Botzung A, Rauch L, Jung B (2020). Differential diagnostic value of total alpha-synuclein assay in the cerebrospinal fluid between Alzheimer’s disease and dementia with Lewy bodies from the prodromal stage. Alzheimers Res Ther.

[8] Fereshtehnejad SM, Yao C, Pelletier A, Montplaisir JY, Gagnon JF, Postuma RB (2019). Evolution of prodromal Parkinson’s disease and dementia with Lewy bodies: a prospective study. Brain.

[9] Kemp J, Philippi N, Phillipps C, Botzung A, Blanc F (2017). Cognitive profile in prodromal disease (dementia) with Lewy bodies. Geriatr Psychol Neuropsychiatr Vieil.

[10] Kemp J, Philippi N, Phillipps C, Demuynck C, Albasser T, Martin-Hunyadi C, Schmidt-Mutter C, Cretin B, Blanc F (2017). Cognitive profile in prodromal dementia with Lewy bodies. Alzheimers Res Ther.

[11] Ferman TJ, Smith GE, Kantarci K, Boeve BF, Pankratz VS, Dickson DW, Graff-Radford NR, Wszolek Z, Van Gerpen J, Uitti R (2013). Nonamnestic mild cognitive impairment progresses to dementia with Lewy bodies. Neurology.

[12] Bousiges O, Bombois S, Schraen S, Wallon D, Quillard MM, Gabelle A, Lehmann S, Paquet C, Amar-Bouaziz E, Magnin E (2018). Cerebrospinal fluid Alzheimer biomarkers can be useful for discriminating dementia with Lewy bodies from Alzheimer’s disease at the prodromal stage. J Neurol Neurosurg Psychiatry.

[13] Blanc F, Colloby SJ, Philippi N, de Petigny X, Jung B, Demuynck C, Phillipps C, Anthony P, Thomas A, Bing F (2015). Cortical thickness in dementia with Lewy bodies and Alzheimer’s disease: a comparison of prodromal and dementia stages. PloS One.

[14] Blanc F, Colloby SJ, Cretin B, de Sousa PL, Demuynck C, O’Brien JT, Martin-Hunyadi C, McKeith I, Philippi N, Taylor JP (2016). Grey matter atrophy in prodromal stage of dementia with Lewy bodies and Alzheimer’s disease. Alzheimers Res Ther.

[15] Thomas AJ, Donaghy P, Roberts G, Colloby SJ, Barnett NA, Petrides G, et al. Diagnostic accuracy of dopaminergic imaging in prodromal dementia with Lewy bodies. Psychol Med. 2019;49(3):396-402. 10.1017/S0033291718000995. 10.1017/S0033291718000995 PMC6331684 29692275

[16] Dufouil C, Dubois B, Vellas B, Pasquier F, Blanc F, Hugon J, Hanon O, Dartigues JF, Harston S, Gabelle A (2017). Cognitive and imaging markers in non-demented subjects attending a memory clinic: study design and baseline findings of the MEMENTO cohort. Alzheimers Res Ther.

[17] Ferman T, Smith G, Boeve B, Ivnik R, Petersen R, Knopman D, Graff-Radford N, Parisi J, Dickson D (2004). DLB fluctuations specific features that reliably differentiate DLB from AD and normal aging. Neurology.

[18] Walker MP, Ayre GA, Cummings JL, Wesnes K, McKeith IG, O'Brien JT, et al. The Clinician Assessment of Fluctuation and the One Day Fluctuation Assessment Scale. Two methods to assess fluctuating confusion in dementia. Br J Psychiatry. 2000;177:252-6. 10.1192/bjp.177.3.252 11040887

[19] Fenelon G, Soulas T, Zenasni F (2010). Cleret de Langavant L: The changing face of Parkinson’s disease-associated psychosis: a cross-sectional study based on the new NINDS-NIMH criteria. Mov Disord.

[20] Gjerstad MD, Boeve B, Wentzel-Larsen T, Aarsland D, Larsen JP (2008). Occurrence and clinical correlates of REM sleep behaviour disorder in patients with Parkinson’s disease over time. J Neurol Neurosurg Psychiatry.

[21] Operto G, Chupin M, Batrancourt B, Habert MO, Colliot O, Benali H, et al. CATI Consortium. CATI: A Large Distributed Infrastructure for the Neuroimaging of Cohorts. Neuroinformatics. 2016;14(3):253-64. 10.1007/s12021-016-9295-8. 10.1007/s12021-016-9295-8 27066973

[22] Petersen RC (2004). Mild cognitive impairment as a diagnostic entity. J Internal Med.

[23] Peres K, Helmer C, Amieva H, Matharan F, Carcaillon L, Jacqmin-Gadda H, Auriacombe S, Orgogozo JM, Barberger-Gateau P, Dartigues JF (2011). Gender differences in the prodromal signs of dementia: memory complaint and IADL-restriction. a prospective population-based cohort. J Alzheimers Dis.

[24] Dumurgier J, Schraen S, Gabelle A, Vercruysse O, Bombois S, Laplanche J-L, Peoc’h K, Sablonnière B, Kastanenka KV, Delaby C (2015). Cerebrospinal fluid amyloid-β 42/40 ratio in clinical setting of memory centers: a multicentric study. Alzheimer’s Res Ther.

[25] Chupin M, Hammers A, Liu RS, Colliot O, Burdett J, Bardinet E, Duncan JS, Garnero L, Lemieux L (2009). Automatic segmentation of the hippocampus and the amygdala driven by hybrid constraints: method and validation. Neuroimage.

[26] Samaille T, Fillon L, Cuingnet R, Jouvent E, Chabriat H, Dormont D, Colliot O, Chupin M (2012). Contrast-based fully automatic segmentation of white matter hyperintensities: method and validation. PloS One.

[27] Mangin JF, Jouvent E, Cachia A (2010). In-vivo measurement of cortical morphology: means and meanings. Curr Opin Neurol.

[28] Langbaum JBS, Chen K, Lee W, Reschke C, Bandy D, Fleisher AS, Alexander GE, Foster NL, Weiner MW, Koeppe RA (2009). Categorical and correlational analyses of baseline fluorodeoxyglucose positron emission tomography images from the Alzheimer’s Disease Neuroimaging Initiative (ADNI). NeuroImage.

[29] Habert M-O, Bertin H, Labit M, Diallo M, Marie S, Martineau K, Kas A, Causse-Lemercier V, Bakardjian H, Epelbaum S (2018). Evaluation of amyloid status in a cohort of elderly individuals with memory complaints: validation of the method of quantification and determination of positivity thresholds. Ann Nuclear Med.

[30] Jellinger KA, Attems J (2011). Prevalence and pathology of dementia with Lewy bodies in the oldest old: a comparison with other dementing disorders. Dement Geriatr Cogn Disord.

[31] Ferman TJ, Boeve BF, Smith GE, Lin SC, Silber MH, Pedraza O, Wszolek Z, Graff-Radford NR, Uitti R, Van Gerpen J (2011). Inclusion of RBD improves the diagnostic classification of dementia with Lewy bodies. Neurology.

[32] Ruiz M, Arias A, Sanchez-Llanos E, Gil MP, Lopez-Ortega R, Dakterzada F, Purroy F, Pinol-Ripoll G (2018). Minor hallucinations in Alzheimer’s disease. J Alzheimers Dis.

[33] McKeith I, Taylor JP, Thomas A, Donaghy P, Kane J (2016). Revisiting DLB diagnosis: a consideration of prodromal DLB and of the diagnostic overlap with Alzheimer disease. J Geriatr Psychiatry Neurol.

[34] Blanc F, Longato N, Jung B, Kleitz C, Di Bitonto L, Cretin B, Collongues N, Sordet C, Fleury M, Poindron V (2013). Cognitive dysfunction and dementia in primary Sjögren’s syndrome. ISRN Neurol.

[35] Chiba Y, Fujishiro H, Iseki E, Ota K, Kasanuki K, Hirayasu Y, et al. Retrospective survey of prodromal symptoms in dementia with Lewy bodies: comparison with Alzheimer's disease. Dement Geriatr Cogn Disord. 2012;33(4):273-81. 10.1159/000339363 22722638

[36] Postuma RB. Prodromal Parkinson disease: do we miss the signs? Nat Rev Neurol. 2019;15(8):437-438. 10.1038/s41582-019-0215-z. 10.1038/s41582-019-0215-z 31152151

[37] Siproudhis L, Pigot F, Godeberge P, Damon H, Soudan D, Bigard MA (2006). Defecation disorders: a French population survey. Dis Colon Rectum.

[38] Barrell K, Bureau B, Turcano P, Phillips GD, Anderson JS, Malik A, Shprecher D, Zorn M, Zamrini E, Savica R (2018). High-order visual processing, visual symptoms, and visual hallucinations: a possible symptomatic progression of Parkinson’s disease. Front Neurol.

[39] Ferman TJ, Smith GE, Kantarci K, Boeve BF, Pankratz VS, Dickson DW, et al. Nonamnestic mild cognitive impairment progresses to dementia with Lewy bodies. Neurology. 2013;81(23):2032-8. 10.1212/01.wnl.0000436942.55281.47 PMC3854825 24212390

[40] Beach TG, Adler CH, Lue L, Sue LI, Bachalakuri J, Henry-Watson J, Sasse J, Boyer S, Shirohi S, Brooks R (2009). Unified staging system for Lewy body disorders: correlation with nigrostriatal degeneration, cognitive impairment and motor dysfunction. Acta Neuropathol.

[41] Roquet D, Noblet V, Anthony P, Philippi N, Demuynck C, Cretin B, Martin-Hunyadi C (2017). Loureiro de Sousa P, Blanc F: Insular atrophy at the prodromal stage of dementia with Lewy bodies: a VBM DARTEL study. Sci Rep.

